# Pulmonary syphilis mimicking lung metastasis: a case report

**DOI:** 10.1097/MS9.0000000000003836

**Published:** 2025-09-02

**Authors:** Virgile Caban, Nathalie Henry, Julie Tronchetti, Jean-Baptiste Lovato, Hervé Dutau, Philippe Astoul

**Affiliations:** a Department of Thoracic Oncology, Pleural Diseases and Interventional Pulmonology, Hôpital Nord, Marseille, France; b Aix-Marseille University, Marseille, France

**Keywords:** case report, HIV infection, lung nodule, secondary syphilis

## Abstract

**Introduction:**

Syphilis, due to *Treponema pallidum*, has various clinical presentations, in particular in patients who are tested positive for HIV, and the nickname of “the great imitator” is very well deserved.

**Case report:**

A 54-year-old HIV-positive man was referred for multiple subpleural bilateral lung nodules without mediastinal lymphadenopathy on a computedtomography (CT) of the chest carried out for symptoms combining dry cough, sweats, night chills, right basi-thoracic pain, and a weight loss of 4 kg in a few weeks. His medical history revealed pneumocystis infection and secondary syphilis, occurring 10 and 8 years ago, respectively. His regular partner did not have any symptoms and deranged biological analysis. The clinical examination was normal except for a maculopapular rash on the back, hands, and forearms. A skin biopsy was performed which revealed the presence of *T. Pallidum*. A CT-guided percutaneous needle aspiration of a pulmonary nodule met the diagnosis of necrotic inflammatory granuloma. A single dose of benzathine penicillin 2.4 million units led to the negative conversion of Venereal Disease Research Laboratory test and ELISA antitreponema antibody index, and the CT scan carried out 3 months later showed a quasi-resolution of several lung nodules.

**Discussion:**

Pulmonary involvement in secondary syphilis is very rare in comparison to congenital or tertiary syphilis. However coinfection with HIV increases the frequency of atypical presentation of the disease. Despite the presence of usual clinical criteria for the diagnosis of pulmonary involvement and an HIV disease controlled with antiretroviral treatment, a CT-guided fine needle aspiration of a nodule was done to rule out neoplastic disease or other infection.

**Conclusion:**

With the rise of syphilis cases, physicians have to be aware of non-specific clinical presentation of the disease in particular in case of coinfection with HIV.

## Introduction

Syphilis, caused by *Treponema pallidum*, remains a significant public health challenge worldwide, with a resurgence of cases in recent years, particularly among high-risk populations such as men who have sex with men (MSM), people living with HIV (PLWH), and individuals who inject drugs. Historically known as “the great imitator,” syphilis presents with a wide range of clinical manifestations, often making diagnosis difficult, especially in its secondary and tertiary stages^[1]^. While syphilis is primarily known for its cutaneous and systemic involvement, pulmonary syphilis represents a rare and often underrecognized form of the disease. The presentation can be highly variable, ranging from asymptomatic cases to severe respiratory symptoms such as cough, dyspnea, pleuritic chest pain, and fever^[2,3]^. Radiological findings frequently include multiple bilateral pulmonary nodules, often necrotic, and ground-glass opacities, which can mimic other infectious and non-infectious pulmonary diseases such as tuberculosis, fungal infections, metastatic malignancies, and inflammatory lung conditions^[4]^. One of the key challenges in diagnosing pulmonary syphilis lies in differentiating it from other more common causes of pulmonary nodular disease. Here we report, using the SCARE guideline^[5]^, a case of an HIV patient presenting with lung nodules diagnosed with secondary syphilis in our academic institution.

**HIGHLIGHTS**

- Syphilis is rising and doctors need to familiarize with uncommon presentations.
- The presence of pulmonary nodules caused by secondary syphilis is a rare diagnosis.
- Clinical and chest radiological presentations in secondary syphilis are non-specific.
- Recognizing pulmonary syphilis particularly in HIV patients is crucial.
- Benzathine penicillin G is the treatment of choice for secondary pulmonary syphilis.

## Case description

**Figure 1.**
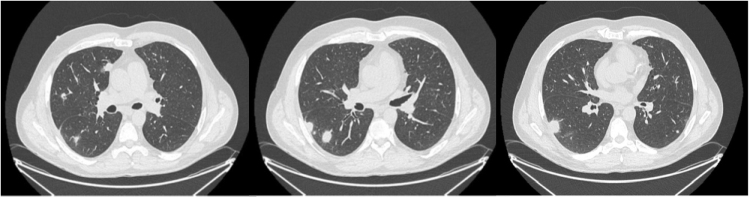
Patient’s chest CT-scan – first consultation.

**Figure 2.**
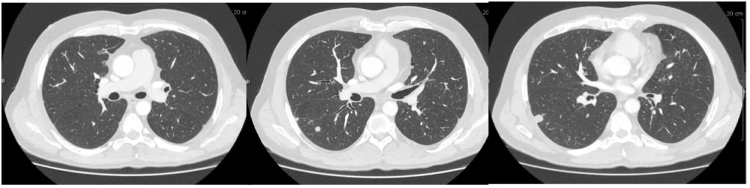
Patient’s chest CT scan 2 months after the unique intramuscular injection of benzathine penicillin G (2.4 million units).

A 54-year-old HIV-positive man, well controlled (CD4 >600 cells/mm^3^ and HIV viral load <2 cp/ml) on Truvada (emtricitabine/tenofovir disoproxil fumarate – 200 mg/245 mg orally twice per day) and Isentress (raltegravir – 400 mg orally twice per day) with no adverse effects, was referred for multiple subpleural bilateral lung nodules without mediastinal lymphadenopathy on a chest CT carried out for symptoms combining dry cough, sweats, night chills, right basi-thoracic pain, and a weight loss of 4 kg in a few weeks (Fig. 1). His medical history revealed pneumocystis infection and secondary syphilis, occurring 10 and 8 years ago, respectively. His regular partner did not have any symptoms and deranged biological analysis. The clinical examination was normal except for a maculopapular rash on the back, hands, and forearms. A skin biopsy was performed showed psoriasiform epidermal hyperplasia, including parakeratosis, elongation of the rete ridges, and spongiosis with neutrophils suggesting *Roseola Syphilitica* and polymerase chain reaction (PCR) revealed the presence of *T. pallidum*. Initial blood test examination showed a normal white blood cell count, an increase of C-reactive protein 126.4 mg/L, normal liver enzymes with aspartate transaminase 31 UI/L, alanine transaminase 47 UI/L, normal total bilirubin and alkaline phosphatase, and elevation of Ɣ-glutamyl transferase 138 U/L. Serology for syphilis was done and Venereal Disease Research Laboratory (VDRL) and *T. pallidum* Hemagglutination Assay turned out positive. Due to the presence of multiple pulmonary nodules, an ambulatory CT-guided percutaneous needle aspiration of a pulmonary nodule was performed to rule out infectious and non-infectious disease, in particular neoplastic lesions, allowing the diagnosis of necrotic inflammatory granuloma. The final decision was to treat the syphilis infection and follow the patient with a CT scan. A single dose of benzathine penicillin 2.4 million units led to the negative conversion of VDRL test and ELISA antitreponema antibody index and the CT scan carried out 2 months later showed a quasi-resolution of several lung nodules (Fig. 2).

## Discussion

From the literature search using “pulmonary syphilis” and “HIV patient” as search terms through PubMed database, pulmonary involvement in secondary syphilis was first described in the late 19th century and remains an uncommon entity with only a limited number of cases reported in the literature^[6,7]^. Pulmonary syphilis, though rare, should be considered in the differential diagnosis of atypical pneumonias and nodular lung diseases, particularly in patients belonging to high-risk groups such as MSM and HIV patients^[3]^. One of the most significant challenges associated with this condition is its highly variable presentation. Patients may present with mild respiratory symptoms or, in some cases, remain entirely asymptomatic, with pulmonary nodules detected incidentally on imaging. In contrast, others may experience significant respiratory distress, fever, and systemic symptoms resembling other infectious diseases, leading to potential misdiagnosis^[8]^. Radiologically, pulmonary syphilis often presents with multiple nodular lesions, which may be necrotic or cavitated, a pattern that can mimic a broad spectrum of pulmonary pathologies, including tuberculosis, histoplasmosis, septic emboli, or even metastatic cancer. In fact, the presence of pulmonary nodules in a patient with a history of syphilis should raise suspicion, particularly when accompanied by a characteristic maculopapular rash, mucosal lesions, or signs of systemic syphilitic involvement such as hepatitis. Studies have shown that pulmonary nodules are the most frequent imaging finding in pulmonary syphilis, with over 70% of cases demonstrating such lesions. Additionally, some cases may exhibit ground-glass opacities or pleural effusion, further complicating the differential diagnosis. Histopathologically, lung biopsies in pulmonary syphilis may reveal granulomatous inflammation with lymphoplasmacytic infiltration, a feature also observed in other infectious and inflammatory conditions^[9]^. *T. pallidum* may occasionally be identified using immunohistochemistry or PCR testing, but its detection in lung tissue is often challenging due to the low bacterial load^[10]^. Therefore, serological testing remains the primary diagnostic tool, with a high index of suspicion needed in appropriate clinical settings. Treatment of pulmonary syphilis follows the standard protocol for secondary syphilis, with intramuscular benzathine penicillin G (2.4 million units) as the first-line therapy. In some cases, ceftriaxone or doxycycline may be used as alternative regimens, particularly in patients with penicillin allergies or in situations where neurosyphilis cannot be excluded. Most patients respond rapidly to treatment, with significant improvement in symptoms and radiological findings within weeks. However, delayed diagnosis may result in unnecessary invasive investigations, prolonged hospital stays, and increased patient morbidity. The association between pulmonary syphilis and HIV co-infection is another critical aspect to consider^[3,11]^. Syphilis has been shown to exhibit more aggressive and atypical presentations in immunocompromised individuals, possibly due to altered immune responses. HIV-positive patients may present with larger or more extensive pulmonary lesions, prolonged disease courses, or coexisting extrapulmonary syphilitic manifestations such as neurosyphilis or ocular syphilis. Given these factors, a lower threshold for syphilis testing should be maintained in PLWH presenting with pulmonary symptoms of unknown origin. From a public health perspective, the increasing incidence of syphilis worldwide underscores the importance of clinician awareness regarding its atypical presentations^[12]^. Improved screening efforts, especially in high-risk populations, may aid in earlier detection and treatment, potentially reducing the burden of severe complications such as pulmonary involvement^[13]^. Furthermore, healthcare providers should be vigilant when evaluating patients with undiagnosed pulmonary nodular disease, particularly when standard infectious and oncological workups fail to identify a definitive cause.

## Conclusion

Despite its rare occurrence, recognizing pulmonary syphilis is crucial, particularly in immunocompromised patients such as those with HIV, where the clinical presentation may be atypical or more severe. Early diagnosis is essential, as delayed recognition can lead to unnecessary invasive procedures and prolonged morbidity. Fortunately, the disease remains highly responsive to penicillin-based therapy, with most patients experiencing complete resolution of both clinical and radiological manifestations.

## Data Availability

Datasets generated during and/or analyzed during the current study are publicly available, available upon reasonable request.
